# Supplementary material for: The Mantle Transcriptome of Chamelea gallina (Mollusca: Bivalvia) and Shell Biomineralization
Source: Animals (Basel). 2022 May 6;12(9):1196. doi: 10.3390/ani12091196 (PMC9100110; doi:10.3390/ani12091196)
Supplement: Supplementary file 1 [file animals-12-01196-s001.zip › animals-1679898-supplementary/SupplementaryMaterial/Tables/SupplementaryTableS1.pdf]

Supplementary Tables S1. Summary statistics of *Chamelea gallina* mantle transcriptome assembly, quality assessment, and differential expression analysis.

- Mantle transcriptome sequencing report and reads trimming

	**S1**	**S2**	**S3**	**SM1**	**SM2**	**SM3**
Total raw reads	47,739,560	51,088,924	49,821,466	58,129,840	49,994,598	50,797,182
Phred≥Q30 (%)	93.87	93.83	94.12	93.47	93.52	93.19
GC (%)	43.55	43.3	43.08	43.54	44	44.2
Cleaned reads (after trimming)	44,708,546	48,441,290	47,371,234	54,383,456	46,642,426	47,003,834

- Cleaned reads assembly using Trinity v2.12

**Trinity Assembly**	
Total transcripts	46,293
%GC	37.88%
N50	452
Min length (bp)	201
Max length (bp)	30,079
Average length (bp)	436.27

- BUSCO (v5.0.0) short summary

	**n**	**%**
Complete and single-copy (S)	4,090	77.2
Complete and duplicated (D)	281	5.3
Fragmented (F)	193	3.6
Missing (M)	731	13.9
Total BUSCO groups searched	5,295	82.5

- Differential Expression analysis (test vs control: Senigallia (S) vs Silvi Marina (SM)) performed with CLC genomics workbench v12.0.

**Differentially Expressed Genes (DEGs)**	**total**	**upregulated**	**downregulated**
FDR <0.05, fold change >4	364	95	269
