# Supplementary material for: The Mantle Transcriptome of Chamelea gallina (Mollusca: Bivalvia) and Shell Biomineralization
Source: Animals (Basel). 2022 May 6;12(9):1196. doi: 10.3390/ani12091196 (PMC9100110; doi:10.3390/ani12091196)
Supplement: Supplementary file 1 [file animals-12-01196-s001.zip › animals-1679898-supplementary/SupplementaryMaterial/Tables/SupplementaryTableS4.pdf]

SENIGALLIA (S)	Temperature	Salinity	Chlorophyll
Sep	25.04 (1.62)	37.12 (0.07)	0.33 (0.16)
Oct	20.54 (0.87)	37.41 (0.16)	0.43 (0.15)
Nov	16.90 (1.43)	37.43 (0.18)	0.61 (0.35)
Dec	0.33 (0.16)	36.39 (0.45)	1.21 (0.46)
Jan	10.64 (0.25)	36.25 (0.17)	1.47 (0.69)
Feb	11.04 (0.54)	35.86 (0.33)	0.73 (0.25)
Mar	11.90 (0.42)	36.82 (0.16)	0.69 (0.31)
Apr	13.61 (1.28)	36.47 (0.27)	0.62 (0.22)
May	17.93 (1.55)	37.15 (0.47)	0.36 (0.13)
Jun	21.49 (1.14)	37.14 (0.14)	0.32 (0.14)
Jul	25.86 (0.61)	36.58 (0.16)	0.29 (0.14)
Aug	27.48 (0.41)	36.88 (0.23)	0.23 (0.13)
Sep	25.00 (0.98)	37.32 (0.04)	0.34 (0.21)
Autumn	17.78 (3.48)	37.20 (0.44)	0.64 (0.41)
Winter	11.24 (0.72)	36.21 (0.40)	1.05 (0.60)
Spring	16.47 (3.45)	36.92 (0.45)	0.48 (0.24)
Summer	26.03 (1.39)	36.89 (0.32)	0.27 (0.14)

Supplementary Table S4. Monthly and seasonally based mean values (values in parentheses represent the standard deviation) of temperature [°C], salinity [PSU] and chlorophyll-a [mg/m^3] at the S sampling site from September 2019 to September 2020.
