# Supplementary material for: The Mantle Transcriptome of Chamelea gallina (Mollusca: Bivalvia) and Shell Biomineralization
Source: Animals (Basel). 2022 May 6;12(9):1196. doi: 10.3390/ani12091196 (PMC9100110; doi:10.3390/ani12091196)
Supplement: Supplementary file 1 [file animals-12-01196-s001.zip › animals-1679898-supplementary/SupplementaryMaterial/Tables/SupplementaryTableS5.pdf]

SILVI MARINA(SM)	Temperature	Salinity	Chlorophyll
Sep	25.41(1.39)	37.57(0.06)	0.23(0.08)
Oct	21.38(0.99)	37.91(0.09)	0.27(0.10)
Nov	17.24(1.49)	38.01(0.03)	0.51(0.33)
Dec	13.22(0.84)	37.83(0.20)	1.06(0.37)
Jan	10.38(0.43)	37.10(0.17)	0.82(0.24)
Feb	11.12(0.44)	36.96(0.02)	0.54(0.20)
Mar	12.26(0.46)	37.10(0.16)	0.42(0.26)
Apr	14.32(1.59)	37.02(0.12)	0.73(0.27)
May	19.34(1.05)	37.28(0.05)	0.39(0.19)
Jun	22.71(1.30)	37.54(0.08)	0.29(0.13)
Jul	26.58(0.57)	37.56(0.13)	0.26(0.13)
Aug	28.21(0.59)	37.63(0.16)	0.24(0.12)
Sep	25.48(1.08)	37.90(0.08)	0.31(0.15)
Autumn	18.38(3.61)	37.93(0.13)	0.54(0.42)
Winter	11.27(0.94)	37.13(0.21)	0.67(0.29)
Spring	17.51(3.69)	37.21(0.23)	0.49(0.30)
Summer	26.76(1.34)	37.66(0.17)	0.25(0.12)

Supplementary Table S5. Monthly and seasonally based mean values (values in parentheses represent the standard deviation) of temperature [°C], salinity [PSU] and chlorophyll-a [mg/m^3] at the SM sampling site from September 2019 to September 2020.
