# Supplementary material for: The Mantle Transcriptome of Chamelea gallina (Mollusca: Bivalvia) and Shell Biomineralization
Source: Animals (Basel). 2022 May 6;12(9):1196. doi: 10.3390/ani12091196 (PMC9100110; doi:10.3390/ani12091196)
Supplement: Supplementary file 1 [file animals-12-01196-s001.zip › animals-1679898-supplementary/SupplementaryMaterial/Tables/SupplementaryTableS6.pdf]

Supplementary Table S6. P-values obtained from the two-way ANOVA for the comparisons of the seasonal temperature [°C], salinity [PSU] and chlorophyll concentration [mg/m^3] between the study areas. ns (not significant) = $P \geq 0.01$; * = $0.01 > P \geq 0.001$; **= $0.001 > P \geq 0.0001$.

**Factor**	**Temperature**	**Salinity**	**Chlorophyll**
season	**	ns	ns
station	ns	ns	ns
interaction	ns	**	**
