# Supplementary material for: The Mantle Transcriptome of Chamelea gallina (Mollusca: Bivalvia) and Shell Biomineralization
Source: Animals (Basel). 2022 May 6;12(9):1196. doi: 10.3390/ani12091196 (PMC9100110; doi:10.3390/ani12091196)
Supplement: Supplementary file 1 [file animals-12-01196-s001.zip › animals-1679898-supplementary/SupplementaryMaterial/Tables/SupplementaryTableS7.pdf]

	Senigallia (S)					Silvi Marina (SM)			
**Temperature**	W	Sp	Su	Au	**Temperature**	W	Sp	Su	Au
W					W				
Sp	**				Sp	**			
Su	**	**			Su	**	**		
Au	**	ns	**		Au	**	ns	**	
**Salinity**	W	Sp	Su	Au	**Salinity**	W	Sp	Su	Au
W					W				
Sp	**				Sp	ns			
Su	**	ns			Su	**	**		
Au	**	**	**		A	**	**	**	
**Chlorophyll**	W	Sp	Su	Au	**Chlorophyll**	W	Sp	Su	Au
W					W				
Sp	**				Sp	ns			
Su	**	*			Su	**	**		
Au	**	ns	**		Au	ns	ns	**	

Supplementary Table S7. Results from Tukey's test pairwise comparison among seasons (W: winter, Sp: spring, Su: summer, Au: autumn) at the sampling stations of S (left columns) and SM (right columns). ns (not significant) = $P \geq 0.01$; * = $0.01 > P \geq 0.001$; ** = $0.001 > P \geq 0.0001$.
