# Supplementary material for: The Mantle Transcriptome of Chamelea gallina (Mollusca: Bivalvia) and Shell Biomineralization
Source: Animals (Basel). 2022 May 6;12(9):1196. doi: 10.3390/ani12091196 (PMC9100110; doi:10.3390/ani12091196)
Supplement: Supplementary file 1 [file animals-12-01196-s001.zip › animals-1679898-supplementary/SupplementaryMaterial/Tables/SupplementaryTableS8.pdf]

**Temperature**		**Senigallia (S)**			
		Winter	Spring	Summer	Autumn
**Silvi Marina (SM)**	Winter	ns			
	Spring		ns		
	Summer			ns	
	Autumn				ns
**Salinity**		**Senigallia (S)**			
		Winter	Spring	Summer	Autumn
**Silvi Marina (SM)**	Winter	**			
	Spring		**		
	Summer			**	
	Autumn				**
**Chlorophyll**		**Senigallia (S)**			
		Winter	Spring	Summer	Autumn
**Silvi Marina (SM)**	Winter	**			
	Spring		ns		
	Summer			ns	
	Autumn				ns

Supplementary Table S8. P-values obtained from the Tukey's test for the comparisons of the seasonal temperature [°C], salinity [PSU] and chlorophyll concentration [mg/m^3] between the study areas. ns (not significant) = P ≥ 0.01; * = 0.01 > P ≥ 0.001; **= 0.001 > P ≥ 0.0001.
