# Supplementary figures and images for: The Mantle Transcriptome of Chamelea gallina (Mollusca: Bivalvia) and Shell Biomineralization
Source: Animals (Basel). 2022 May 6;12(9):1196. doi: 10.3390/ani12091196 (PMC9100110; doi:10.3390/ani12091196)

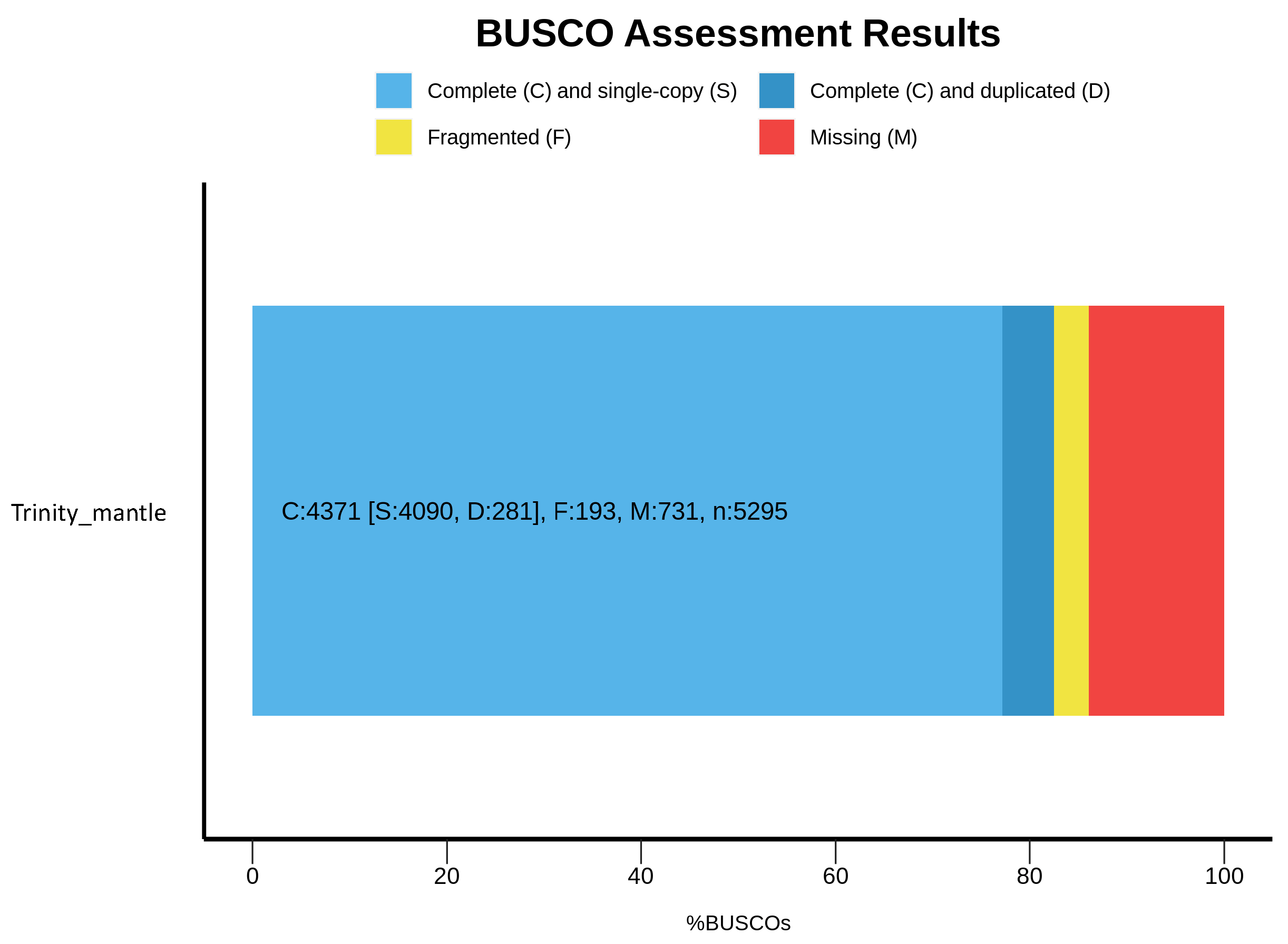

Supplement: Supplementary file 1 [file animals-12-01196-s001.zip › animals-1679898-supplementary/SupplementaryMaterial/Figures/SupplementaryFigureS1.tif]

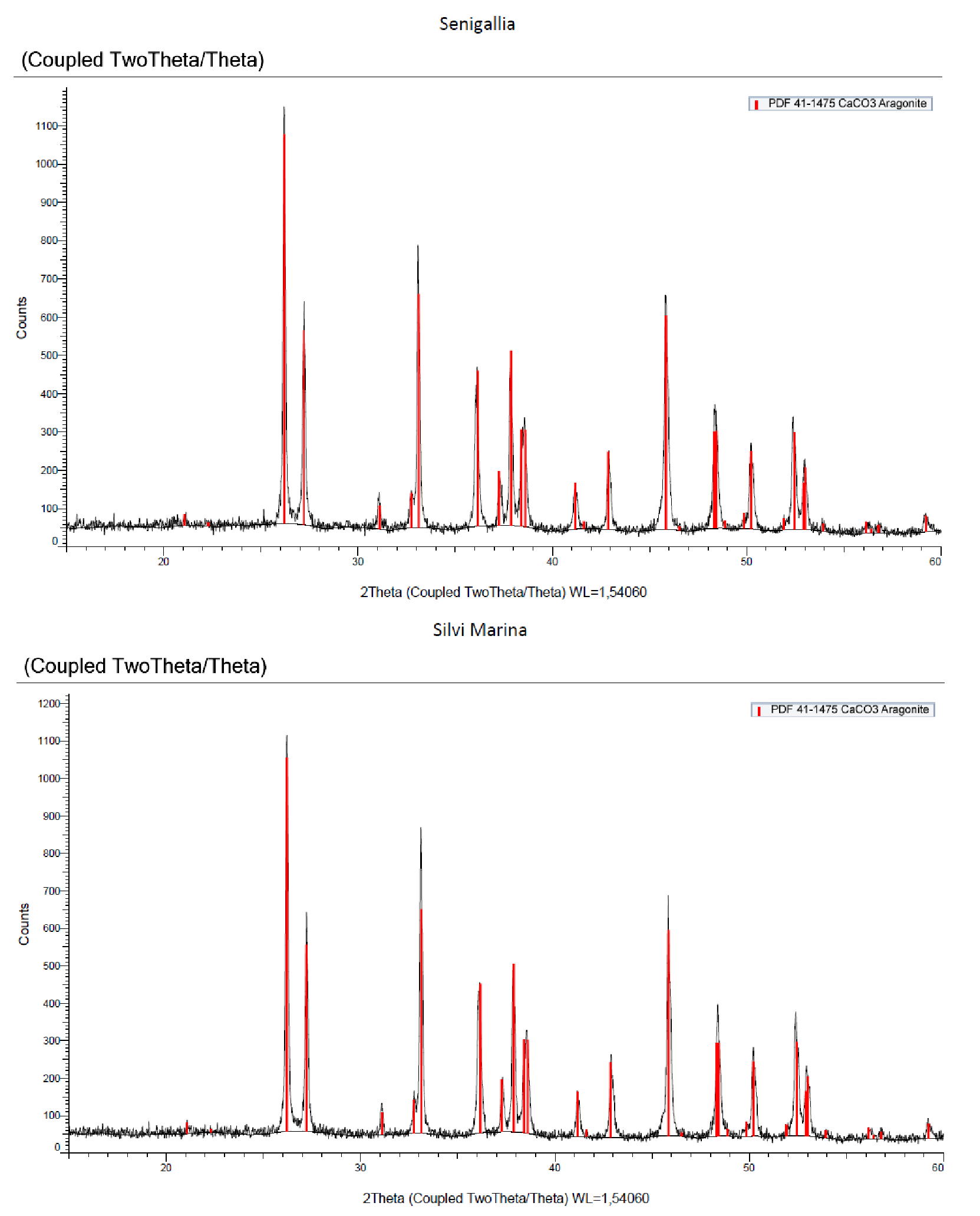

Supplement: Supplementary file 1 [file animals-12-01196-s001.zip › animals-1679898-supplementary/SupplementaryMaterial/Figures/SupplementaryFigureS2.tif]
